# The Impact of Vacant and Abandoned Property on Health and Well-Being: A Qualitative Inquiry

**DOI:** 10.1007/s11482-024-10325-w

**Published:** 2024-06-11

**Authors:** Gabriella D. Roude, Kimberly Wu, Lisa Richardson, Amber Tucker, Lolita Moss, Michelle Kondo, Christopher N. Morrison, Charles C. Branas, Jeanette Gustat, Katherine P. Theall

**Affiliations:** 1 Institute of Women & Ethnic Studies, New Orleans, LA USA; 2 School of Public Health & Tropical Medicine, Tulane University, New Orleans, LA USA; 3 USDA Forest Service, Northern Research Station, Philadelphia, PA USA; 4 Mailman School of Public Health, Department of Epidemiology, Columbia University, New York, NY USA; 5 Department of Epidemiology and Preventive Medicine, School of Public Health and Preventive, Medicine, Monash University, Melbourne, Australia; 6 Tulane Mary Amelia Center for Women’s Health Equity Research, New Orleans, LA USA; 7 Tulane Violence Prevention Institute, New Orleans, LA USA

**Keywords:** Neighborhood characteristics, Neglected property remediation, Physical environment, Sense of safety, Violence

## Abstract

This qualitative study explored the role of neglected properties and neighborhood environment characteristics on a sample of New Orleans, Louisiana residents’ health and well-being, sense of community, sense of safety, and civic engagement. We hypothesized that residents would identify conditions of their neighborhood's physical environment, including neglected properties, as one factor that impacted their health and other aspects of well-being. Seventy-four (N = 74) participants, including women, men, youth, young adults, and community leaders, took part in 11 focus groups (n = 51) and 23 key informant interviews. Thematic content analysis through inductive and deductive coding cycles of interview transcripts revealed five main categories related to urban neighborhood-built and social environments: 1) health and well-being, 2) sense of community, 3) sense of safety, 4) civic engagement, and 5) youth and family violence. Ten themes were developed and included, for example, the role of neighborhoods in delineating access to health-promoting characteristics and resources; the role of neighborhood social networks as crime prevention strategies; resident-led decision-making in neighborhood improvements; the negative impact of neglected properties; and the role of the local government in improving physical infrastructure. These findings affirm that residents were aware of and impacted by the cyclical nature of built environment neglect on health and well-being, community violence, neighborhood cohesion, civic engagement, and youth violence. Participants recommended improving neighborhood conditions to shift resident mindsets about the health of neighborhoods, reduce violence, and improve quality of life.

## Background

United States urban environments contain neighborhoods of concentrated poverty, largely inhabited by racially and ethnically minoritized populations, and are often characterized by unsafe housing, vacant properties, and abandoned structures. Disinvested neighborhoods have few safe, clean, and green spaces for leisure and exercise and suffer from high rates of violent crime, litter, pollution, exposure to environmental toxins, and limited availability of fresh, healthy food sources (Edmonds et al., 2015). Disinvested neighborhoods are also more likely to have underperforming schools and fewer job opportunities, limiting educational, economic, and social mobility (Edmonds et al., 2015). Present-day neighborhood conditions have been shaped by historically-rooted systemic oppression due to policies and practices such as redlining, which have perpetuated a stark racial wealth gap in cities across the U.S. and are linked to health inequities (e.g., life expectancy) and environmental exposures (e.g., air pollution levels) (Graetz and Esposito, 2022; Lane et al., 2022).

While several neighborhood characteristics influence health, there has been increasing attention on the impact of deteriorated neighborhood conditions, such as vacant and abandoned property (South et al., 2023; Sadler et al., 2017). Vacant lots and buildings are visible signs of neighborhood disorder and are correlated with violence, fear, and often have overgrown vegetation and dumping that may provide opportunities for illegal activity, harboring illegal firearms, and misuse of spaces (Disorder and decline: crime and the spiral of decay in American neighborhoods, 1991; Wei et al., 2005; Keizer et al., 2008; Ross and Mirowsky, 1999; LaGrange et al., 1992; C. C. Branas et al., 2016). Such conditions are linked to a range of adverse health outcomes, including poor birth outcomes, stress, mental health, physical activity, heart rate, injury, crime and violence, blood lead levels, insect vectors, and heavy metal contamination (Garvin et al., 2012; Nowak and Giurgescu, 2017; Sivak et al., 2021). New Orleans, Louisiana, has a well-recognized vacant and abandoned property problem, with approximately 22,000 vacant properties as of the first quarter of 2023 (Building Blocks New Orleans, 2023). The city also grapples with elevated violence rates, chronic and infectious conditions, and negative health behaviors (Wilson, 2023; Centers for Disease Control and Prevention, National Center for Chronic Disease Prevention and Health Promotion, Division of Population Health., 2022). Though rich in culture and history, racial inequities are pronounced and pervasive in all aspects of city life, including the education system, neighborhood structure, response to natural disasters, and health care outcomes (Building Blocks New Orleans, 2023; Wilson, 2023; Centers for Disease Control and Prevention, National Center for Chronic Disease Prevention and Health Promotion, Division of Population Health, 2022). New Orleans offers a unique opportunity to gain a deeper understanding of the role of neighborhood conditions, such as vacant and abandoned property, on the health and well-being of residents.

As detailed in the works of McLeroy et al., (1988) and Stokols, (1992), the social-ecological model emphasizes that individual health and behaviors are significantly shaped by various levels of influence, with community factors playing a pivotal role. An ecological framework posits that health promotion should occur through a multidimensional approach ranging from individual to environmental-level measures. The social-ecological model provides a foundation for understanding the role and impact that neighborhood physical and social characteristics have on the quality of life of residents. Similarly, the Robert Wood Johnson Foundation’s Culture of Health Framework outlines four action areas to achieve population health, well-being, and equity (Chandra et al., 2016); we investigate two areas: 1) making health a shared value and 2) creating healthier and more equitable communities through the built environment conditions. Drawing from the social-ecological model and the culture of health framework, in this study we center residents' lived experiences to examine how neighborhood physical and social characteristics influence health and well-being. There is a dearth of knowledge about how residents experience and perceive the interconnectedness between neighborhood conditions and well-being. Understanding *how* residents describe the impacts of their neighborhood environment on health is critical to inform solutions designed to improve population health.

## Method

### Study Design

This qualitative study was part of a parent study called the Healthy Neighborhoods Project (HNP), aimed at remediating vacant properties to reduce violence and improve health. The HNP is a multimethod study designed with a cluster randomized controlled trial and a qualitative component. The longitudinal cohort arm of the HNP followed the experiences of neighborhood residents throughout the 5-year project. Survey participants were recruited from 194 randomly selected clusters (101 intervention and 93 control clusters) using a 1/8-mile radius and in areas of the city with the highest violent crime and poverty rates. Publications of survey data from the parent study are currently being developed. This study provides findings from the qualitative component, including key informant interviews and focus groups conducted between October 2019 and November 2021 with 74 residents. Table 1 includes detailed descriptions of the sample, methods, and format.

**Table 1:** Key Informant & Focus Group Interviews Overview

Population	Number of Focus Groups (# participants)	Number of Key Informant Interviews	Format
Women	3 (n = 12)	23 (13 sectors represented)^1^	28 online 6 in-person
Men	2 (n = 6)		
Youth (< 18 yrs.)	2 (n = 13)		
Young adults (18–25 yrs.)	4 (n = 20)		
Total Participants: 74 Residents from 28 Neighborhoods^2^			

^1^Sectors represented: Law Enforcement (New Orleans Police Department), Violence Prevention, Neighborhood Association, Community Outreach, Land Use/Real Estate/Community Development, City Government, Youth Engagement, Social Work, Health, Education/Higher Ed., Housing, Criminal Justice Reform/Formerly Incarcerated Persons, Faith Based Organization

^2^Neighborhoods represented: Algiers, Bayou St. John, Central City, Desire Area, Dillard, East Riverside, Faubourg Livaudais, Freret, Gentilly, Gentilly Terrace, Hoffman Triangle, Holy Cross, Iberville, Lake Terrace Oaks, Little Woods, Lower Garden District, Lower Ninth Ward, Marigny, Michoud, New Aurora, Old Aurora, Pines Village, Seventh Ward, St. Bernard Area, St. Roch, Treme/Lafitte, Tulane-Gravier, West Lake Forest

A combined methodological orientation of grounded theory and narrative inquiry was adopted to guide our study design and analysis (Lal et al., 2015). Semi-structured interviews were conducted with residents from 28 of New Orleans’ 73 neighborhoods. Eleven focus groups (n = 51) were held with four demographically determined groups, including women, men, youth (age 13–18), and young adults (age 19–25), ranging from two to eight participants per session. Key informant (n = 23) interviews were conducted with community leaders representing 13 professional sectors. Participants were recruited through convenience and snowball sampling through local community-based organizations, workforce development, substance abuse and housing assistance programs, high schools and alternative high schools, the police department, and a violence interruption program. Key informants were contacted directly to solicit interest and participation. All participants received a $25 gift card. Tulane University’s Institutional Review Board approved the study.

### Interview Procedure

Participants completed a demographic survey and provided consent before the interviews. The study team referenced an interview protocol outlining procedures for data collection, data management, and data preparation for analysis throughout the project duration. Two experienced facilitators conducted the interviews; one led the conversations while the other took notes. Eighty-two percent of interviews were conducted online via Zoom™ (Zoom Video Communications, Inc., San Jose, CA) in adherence to COVID-19 social distancing safety mandates. A *“How to Download & Use Zoom”* guide was shared with participants before interviews and technical support was provided as needed. A semi-structured interview guide was utilized to inquire about participants' experiences, attitudes, and beliefs about health and well-being, the role of neighborhood conditions in their lives, descriptions of their present neighborhood-built and social environments, perspectives on community violence, and neighborhood improvements. Three pilot focus groups were conducted to refine the interview guide. Table 2 outlines interview questions. The average duration of key informant interviews was 40 min, whereas the focus groups lasted 60 min on average.

**Table 2:** Key Informant & Focus Group Interview Protocol Questions

Orientation to Health (Definitions and Concepts):
a. What does being healthy mean to you? What would you say contributes to someone’s health?
b. How about well-being? What contributes to someone’s well-being?
c. What would you say contributes to health and well-being in your specific neighborhood? In New Orleans in general?
d. What would you say shows that we value health in our community?
Perceived Roles of Neighborhoods & Conditions:
a. Do you think your neighborhood is safe? Why or why not? What does it mean for you to feel safe in your neighborhood?
b. How does violence (in your community and/or in your household) impact health and well-being? Can you give a specific example?
c. How does your relationship with other people that live in your neighborhood impact your health and well-being, if at all? How about your day-to-day life?
d. How active are the people that live in your neighborhood in improving neighborhood conditions? In what ways do people work to improve neighborhood conditions?
e. What would you change about your neighborhood to make it a healthier place to live?
Connection between violence and neighborhood conditions:
a. In your opinion, how do neighborhood conditions encourage (allows) or discourage (prevents) violence? In the neighborhood? In the home?
b. How do neighborhood conditions influence how safe or unsafe you feel in your neighborhood or other neighborhoods where you spend time?
Sustainability:
a. How can we maintain improvements to your neighborhood? For example, if the city cleaned up an empty lot and mowed the grass, what can they do to make sure it gets done regularly?
b. What kinds of spaces would you like to see empty lots turned into?
c. Is there anything else you want to tell us about living in your neighborhood?

## Results

### Participant Demographics

As shown in Table 3, participant ages ranged from 13–74 years, with nearly half identifying as women (49%, n = 36) and the majority as Black (74%, n = 55). On average, residents had lived at their current address for six years. Over half were renters (54%, n = 40), and almost one-fourth owned their homes (23%, n = 17).

**Table 3:** Demographic Characteristics of Interview Participants (n = 74)

Variable	n (%)
Age	
13–17 years old	13 (18%)
18–24 years old	20 (27%)
25–34 years old	5 (7%)
35–44 years old	9 (12%)
45–54 years old	8 (11%)
55 + years old	13 (18%)
Missing	6 (8%)
Race	
Black/African American	55 (74%)
White	7 (9%)
Asian/Pacific Islander	2 (3%)
Multiracial	4 (5%)
Missing	6 (8%)
Sex/Gender	
Female	36 (49%)
Male	32 (43%)
Non-binary	2 (3%)
Missing	4 (5%)
Employment	
Homemaker	1 (1%)
Employed	30 (40%)
Out of work/searching	7 (9%)
Retired	2 (3%)
Student	8 (11%)
Unable to work	5 (7%)
Missing	7 (9%)
Children (#) Under 18 in Home	
0	36 (48%)
1	9 (12%)
2	11 (15%)
3 +	11 (15%)
Missing	7 (9%)
Years Lived at Current Housing	
< 1 yr	11 (15%)
1–5 yrs	29 (39%)
6–10 yrs	9 (12%)
11–15 yrs	6 (8%)
16 + yrs	6 (8%)
Missing	13 (18%)
Housing	
Rent	40 (54%)
Own	17 (23%)
Shelter/Transitional	11 (15%)
Missing	6 (8%)

### Thematic Analysis

Key informant and focus group interviews were recorded and transcribed verbatim, with transcripts generated via the Zoom transcription feature and edited by trained research team members for accuracy and format. *Atlas.ti* version 8 was used for analysis. The research team coded the data for explicit and implicit content following Vaismoradi et al., (2016b) category and theme development phases. The analysis team consisted of two independent coders (G.R., K.W.) who coded interview transcripts through deductive and inductive coding, labeling text segments based on codes determined in advance as informed by the interview guide questions and allowing new codes to emerge directly from the data through independent line coding. Descriptive codes were organized by categories within a codebook. Coders used analytical memos to document codebook changes and note patterned observations across transcripts (e.g., code co-occurrence, contradiction, similarities). The coders discussed their interpretations and summarization of emergent categories and themes as informed by code definitions, code frequency, code categories, and patterned use of codes. A shared codebook was used and updated throughout an iterative coding process to reduce individual biases and ensure the trustworthiness of the analysis. Continuous discussion of code operationalization, categories, and themes facilitated consensus-building between coders. Five main categories related to the neighborhood environment were identified and included 1) health and well-being, 2) sense of community, 3) sense of safety, 4) civic engagement, and 5) youth and family violence. Ten themes were developed, as depicted in Table 4 and described below.

### Health and Well-being

#### Theme 1: Residents detail a holistic view of health

Residents spoke of health and well-being in terms of physical, mental, emotional, social, spiritual, and environmental factors. Health and well-being were described interchangeably, yet residents often defined well-being as encompassing many aspects versus merely the absence of physical ailments (Table 4). The aggregate of one’s surroundings, including the neighborhood’s social and built environment, was often identified as a significant contributor to health and well-being, as described by an informant in community outreach: *“A healthy environment comes first. Cause whatever you live in that’s kinda what your life is. Safe and non-toxic, I’d say those are the biggest things.”*

#### Theme 2: Neighborhoods delineate access to health-promoting characteristics and resources

Residents perceived their communities as a determining factor for access to health-promoting physical spaces and resources, speaking at length about the implications of proximity to amenities such as greenways and parks that can facilitate physical and social activities, stores to buy quality food, presence of sidewalks, and safe roads (Table 4). Residents also described the importance of access to education, healthcare, financial and employment opportunities. A key informant working in mental health care shared, *“health and well-being is contingent on people having access to resources to have their needs met…I think a lot of how, not so much in my community, because we have hospitals nearby, but there are you know there are areas that are hospital deserts right, just like we have grocery deserts.”*

Residents were attuned to disparities in the *quality* of food outlets, health resources, roads, sidewalks, recreational facilities, and public green spaces across neighborhoods with differing economic and racial components (Table 4), as well as in exposure to community crime and violence. A key informant working in community health shared:

*“So, you know, and even now, today, I just don't feel safe and I will never move in the 7th Ward. I don’t like to frequent the 7th Ward. I don’t like the streets, I don’t park my car around there. You know, I lost two family members in that neighborhood for no reason at all.”*

### Sense of Community

#### Theme 3: Neighborhood interpersonal relationships cultivate a sense of community and support systems

Relationships with neighbors and broader community members were a prevalent theme across interviews. Trusted relationships with neighbors fostered a sense of security; a key informant shared: *“We know our neighbors on the block…there’s a comfort in that, that also might lessen stress levels, it actually lowers my stress seeing my neighbors.”* Others described how neighborhood social bonds were essential to those who required additional social support, including elderly community members, individuals without family nearby, families impacted by COVID-19, and those with children. Collective efficacy and neighborly support were evident in participant descriptions of collecting a neighbor's mail, giving someone a ride, organizing neighborhood parties and meals, and watching for unwanted activity. Residents interact and build shared values in their neighborhoods, allowing for community-based support systems (Table 4).

### Sense of Safety

#### Theme 4: Presence of community crime influences perceptions of personal safety, social engagement, and health

Violence and crime influenced participants’ sense of safety, fear of harm, and hypervigilance and resulted in less engagement with the immediate community. Residents often expressed sentiments such as, *“you got to be aware of your surroundings at all times and that is a stress factor*.” A young adult participant also shared: *“I do get worried about that [crime]…like every time I go out I can’t guarantee that I’ll make it back safely every time.”* Residents described how heightened awareness of crime, such as hearing gunshots, influenced individual behavior. A men’s focus group participant shared:

*“When you hear certain sounds that you feel are unsafe it can also deter people from getting out and just walking and exploring their neighborhoods you know from a recreational standpoint but, as well as the physical fitness standpoint, you know you just feel like you just need to close yourself in your home. And so yeah it plays on the psyche in many different ways.”*

Participants often shared that a lack of safety fostered distrust among community members (Table 4). Additionally, several participants distinguished that youth, women, and gender non-conforming people experienced safety and community engagement differently due to their identity. A youth participant shared, *“I wouldn’t say Canal Street is safe. Not for women at least.”* Another young adult participant shared that safety is the ability to express themselves in their community freely:* “I always felt safe because I could always express myself and who I was, just honor my emotions and my spirituality and myself.”*

#### Theme 5: Neighborhood social networks as crime prevention

Residents described that key individuals in their neighborhood helped buffer the occurrence of crime by having *“eyes on the streets”*, and notifying others of the presence or suspicion of unwanted activity. A key informant working in city government shared: *“I mean you know we-we have these, these Nest cameras or whatnot, but that's not better than the person sitting on their porch across the way right?”* Other methods of community-led surveillance included exchanging contact information, informing others who may not be aware of areas or times of day to avoid outdoor activities, and online and in-person crime watch groups (Table 4).

#### Theme 6: Neglected physical environment conditions negatively impact residents and communities

Residents identified built environment characteristics that impact sense of safety and security. Vacant and abandoned properties were perceived to attract unwanted behaviors and crime. A women’s focus group participant shared, *“…criminalize these vacant lots because it does bring criminal elements into the area.”* A men’s focus group participant expressed how neglected areas can convey and invite disarray: *“When there's a lot of abandoned areas it, it's just you can do whatever you want you know what I mean, crime-wise or whatever.”* A key informant working in neighborhood revitalization shared how neglected areas can result in a snowball effect for illegal dumping:

*“I guess the biggest contributing factor that is all the abandoned buildings, they’re like incubators. Then you know that’s where the big piles of litter start and they spread all over the neighborhood. And after that follows the tires, the abandoned couches. Because people come into a neighborhood looking to dump something, they just look for stuff like that and figure ‘oh there’s a place!’”*

Residents explained that neglected properties have a mental and emotional impact and signal that a community is unhealthy. A social worker key informant shared, *“I would say, mental health, because if you [live] in a neighborhood where people really don't care about their property or their environment, it kind of, it affects you mentally.”* Neglected properties were commonly described as *“eyesores”* and explained that living in a community with significant property neglect *“weighs on you.”* The presence or absence of litter, illegal dumping, and conditions of homes were identified as environmental signals of residents' investment (Table 4). A neighborhood association member spoke to this point: *“…it's an environment where people actually care about the environment, they're constantly picking up debris or leaving their lawns well-manicured you know.”*

### Civic Engagement

#### Theme 7: Residents champion physical environment improvements and sustainability efforts

Residents participate in decision-making processes that shape their communities by joining neighborhood associations, contacting neighborhood representatives, and reporting physical hazards. Residents provided instances where they participated or observed others in their community engage in activities to improve or maintain their immediate physical environment (Table 4). A neighborhood association member shared, *“When I take my early morning walk at six o'clock, I'll bring a little old folks’ basket, as they call it, and I’ll put a bag in and on my trailer, I’ll just pick up the trash…”.*

Several interviewees shared that they believe others in their community are not active, unable to be involved, or are indifferent to the physical environment's appearance and maintenance. A young adult participant elaborates: *“I think that in my neighborhood specifically there’s not a lot of people that really care about the conditions of where we live [that] are able to act in a way that’s helpful down here.”* While a member of a neighborhood association shared that neighborhood betterment efforts encourage others to invest in their immediate environment: “*…there’s ones that really don't care and you're showing why you do care and all of a sudden they care as well.”*

#### Theme 8: Repurpose abandoned lots to address community needs, especially for populations most in need

Residents had many ideas for repurposing vacant parcels to benefit their neighborhood communities, including green spaces, green infrastructure, housing, community centers, and sources of economic opportunities (Table 4). A women’s focus group resident shared, *“get one of these lots and use it as income”* by allowing businesses to be established. A participant in a men’s focus group shared: *“We live in a town where heavy rain events happen so maybe more needs to be done in terms of using the lots for urban water management approaches and installations.”* A lead member of a neighborhood association shared: *“Well, I would get with someone who wants to come in and build houses on them.”*

#### Theme 9: Resident perceptions of the role of government in improving physical infrastructure

Residents described that community members who are active in neighborhood betterment efforts needed more resources, especially in socio-economically disadvantaged areas:

*“Where as neighborhoods like the Lower 9 where people are generally lower income are not going to be as able to contribute financially to making sure that the rest of the neighborhood is up to par or don’t have tons of time because they may be working two or three jobs to call up their various departments and making sure that things get done. So you know that’s really where we need you know the city’s support.”*

In light of challenges present in sustaining lot repurposing efforts, a participant in a women’s focus group shared: *“like some of the vacant lots people want to make like community gardens, but like I said who's going to fund that and not everyone in communities can upkeep it if that is possible.”* While residents contribute to neighborhood upkeep, many expressed that it is ultimately the local government’s responsibility to remediate and maintain larger-scale infrastructure concerns and that city officials were slow to respond to formal complaints about the physical environment (Table 4).

Many residents described that neighborhood infrastructure, such as roads, sidewalks, electricity, and water management, needs to be improved. On neighborhood mobility and accessibility, a key informant shared, “*I find myself having trouble trying to take a walk down the streets.”* Participants described specific neighborhood areas where public infrastructure conditions are currently unsafe for residents, especially during emergency weather events. A key informant working in crime prevention shared:

*“...some of those streets are access points to the interstate. So, you can get out your house, but if you're trying to get on the interstate you stuck because you got to go through flood water, so it kind of puts you in a precarious situation you know.”*

Many residents described the severity of street flooding in their neighborhood, at times damaging property, preventing access to their homes, and affecting their safety. Furthermore, the recurrence of weather-related emergencies such as hurricanes, tornados, and frequent flooding in particularly vulnerable neighborhoods requires preparation and recovery beyond individual residents' purview.

### Youth and Family Violence

#### Theme 10: Positive youth engagement in the home and community as crime reduction and prevention strategies

Several adult participants shared that youth involvement in physical environment improvement and sustainability efforts offers an opportunity for youth leadership and access to economic opportunities and mitigates youth involvement in crime (Table 4). Residents described the importance of youth engagement, especially for preventing crime. Examples of positive youth engagement were described at an interpersonal and community level. Many adult participants explained how family can mediate youth crime involvement: *“I had a lot of family around me…so I think that as a kid, I guess I was shielded from my external environment.”* On the contrary, a few participants described how the family unit might negatively guide youth behavior. To this point, a key informant shared: *“But you know it do start with the kids. As a matter of fact, it start at home, it start at home.”* While another participant in a men’s focus group highlighted how positive youth engagement needs to be actualized at the community level first (Table 4): *“If you got a hostile environment there is no re-teaching the kids…you have to make neighborhoods safe first before you can start doing with these kids.”*

Several adult participants spoke about the types of violence and crime they have witnessed or heard of young people engaged in, including car theft, involvement in gang disputes, use of guns, and robbery. Youth spoke about navigating their environments to manage their safety: *“I would say we’re not even safe because we are next to a police station, cause there’s bars across the street… this area is known for it’s drinking.”* Many participants expressed the need to invest in resources, programs, and relationships that would encourage young people to access healthy options for their development and well-being.

**Table 4:** Themes and Associated Quotations

Categories/Themes:	Quotations/Population:
Health and Well-being	*“A human being is more than just their body, that's why I mentioned the mental, emotional, and the spiritual side of it cause the body part–working out and eating right, that takes care of your physical matter, but there is a part of your matter that’s immaterial that you also need to take care of in order to be stable, in order to be balanced.”-* Young Adult
Theme 1: *A holistic view of health* Theme 2: *Neighborhoods delineate access to health-promoting characteristics and resources*	*“…there’s a stark difference in the quality of life that people have in this city. Like when you say go into New Orleans East I have trouble actually going to that part of the city… And then, when I go somewhere that's like around St. Charles or like on the Upper/Lower Garden District, everything is within walking distance, no matter if it’s niche shops or like things that are essential to people's everyday living, such as having access to doctors, having access to food, art centers and whatnot…”*- Women’s Focus Group
	*“…so I don't live that far from City Park so anytime I want to go there, I can just walk there in less than an hour. In terms of like I would say healthier options of food um probably like probably like a 10 min bus ride or a 20 min walk…”*—Young Adult
Sense of Community Theme 3: *Neighborhood interpersonal relationships cultivate a sense of community and support systems*	*“I think that neighborhoods are safe when people walk through them, they speak to one another, we know one another by name. And I think I’ve made that an agenda of mine. I walk around the neighborhood in a one/two block area and I meet my neighbors and I speak to them, especially the elders because I think, especially since COVID, and Ms. [name redacted] has a stomach ache, Ms. [name redacted] has a knee problem and to me that's kind of what makes the neighborhood a neighborhood.”-* Women’s Focus Group
Sense of Safety	*“When there's you know certain ills, certain aspects of a neighborhood or an area that are visible from an unhealthy standpoint, the crime, the blight, and all of that, to me it sows uneasiness it puts people in a mindset of mistrust, suspicion, blame you know it brings out the biases. And all that is just not healthy you know, if for the most part an area looks good, low crime, and all those other [amenities] and assets are addressed then people feel better towards one another, you know. The environment plays such a huge part on the thinking of people, you know.”-* Men’s Focus Group *“In this town if somebody is up to no good somebody in the neighborhood knows who they are and knows who their family is and quite frankly much much more likely to get some result if you send it through that channel rather than the official channels…Uh and those folks that have been around here for a long long time they’re the engine under that hood when it works.”-* Key Informant, Community Outreach *“When the streets are clean, and when the homes still look pretty new, even if they're old, that means everyone takes care of the things they have. It’s usually a good sign for me.”—*Youth
Theme 4:* Presence of community crime influences perceptions of personal safety, social engagement, and health* Theme 5: *Neighborhood social networks as crime prevention* Theme 6: *Neglected physical environment conditions negatively impacts residents and communities*	
Civic Engagement Theme 7: *Resident-led physical environment improvements and sustainability efforts* Theme 8: *Repurpose abandoned lots to address community needs, especially for populations most in need* Theme 9: *Resident perceptions of the role of government in improving physical infrastructure*	*“somebody’s garbage can ain’t put up or put out there, we’ll put it up for them or put it back on a curb. Different things like that, so they kind of, we keep the community in order and straight.”—*Key Informant, Reform/Formerly Incarcerated *“Center where there is a gym, there's something for everybody's passion, whether it's working out, or art, or music, or anything. A multi-center building where your passions can be worked instead of your frustrations and your issues, so a community center.”*- Young Adult *“I think our neighborhood needs more attention in regards to updating infrastructure…so that includes fixing streets, making sure there is lights everywhere. Making sure that our plumbing system and electrical systems and all of those things are you know are in good repair. Making sure that our storm drains are clear.”*—Key Informant, Land Use/Community Development *“I've written the city about three or four times, because of this sinkage and the clogged drains in my neighborhood, especially in front of my home.”*—Key Informant, Health/Social Work
Youth and Family Violence Theme 10: *Positive youth engagement, in the home and community, as crime reduction and prevention* *“give them [youth] something to do so that they won't have nothing to do on the street. You know occupy them constructively, you know what I’m saying.”* – Men’s Focus Group	*“After school program where children who want to make you know a little extra money can come help in they own neighborhood and clean the blighted lot like teaching them skills and giving them you know, money to do things they want to do where they won't have to be leaded by drug dealers and criminal thing, and you know do this to make money.”-* Key Informant, Reform/Formerly Incarcerated

## Discussion

This qualitative study explored how the neighborhood environment, specifically neglected conditions, impacts resident health, sense of community, safety, and civic engagement as perceived by a sample of 74 residents representing 23 neighborhoods in New Orleans, Louisiana. Ten key themes were identified through an iterative thematic analysis process of the interview data. Our findings corroborate existing research and ecological theories that identify neighborhood environments as pivotal sites for improving various quality of life outcomes for residents (Golden and Earp, 2012; Chandra et al., 2016). This study affirms the principles of the social-ecological model and the culture of health framework, demonstrating that health is influenced at multiple levels, with neighborhood social cohesion and built environment assets as necessary pathways for promoting health equity. The results expand existing theories of neighborhoods and health, particularly with respect to mechanisms that may impact health and violence—social connection and cohesion in particular. Through this study, resident and community leader experiences offer several important insights into the processes through which neighborhood physical and social characteristics are interrelated.

First, the themes *residents detail a holistic view of health* and *neighborhoods delineate access to health-promoting characteristics and resources*, summarize residents' nuanced understanding of the multilevel mechanisms by which their neighborhood context and quality of life are interconnected. Similarly, residents frequently identified the ways that neighborhoods prevent or promote access to essential amenities and health-promoting resources (e.g., grocery stores, hospitals, schools, green spaces, reliable infrastructure) and social assets (e.g., social networks, youth engagement, collective efficacy). Comparably, the works of Satcher, (2022) and Tung et al., (2018) discussed the impacts of spatial inequality on health and how resource scarcity in urban neighborhoods including low access to grocery stores, pharmacies, and fitness resources are associated with decreased resident safety and physical activity. Study participants described how disinvestment in predominantly Black neighborhoods impacts resident well-being, sense of safety and negatively impacts mental health across the life course.

Second, several themes illustrate residents' perceptions regarding the effects of vacant and neglected neighborhood conditions and the need to utilize vacant properties to improve communities. These themes include: *Neglected physical environment conditions negatively impact residents and communities; residents champion physical environment improvements and sustainability efforts; repurpose abandoned lots to address community needs, especially for populations most in need; resident perceptions of the role of government in improving physical infrastructure.* Resident perspectives underscored residents' and city officials' need for neighborhood upkeep, including neglected property remediation, as a pathway for health promotion, violence prevention, a sense of safety, and community engagement. In concert with resident sentiments, the quality and consistency of neighborhood maintenance have been shown to moderate several aspects of health, including stress levels and poor mental health (Garvin et al., 2012; Boardman, 2004). Also, residents identified that physical disorder may result in fewer interpersonal interactions among neighbors, decreasing community collective efficacy.

Several studies have discussed property abandonment and the ensuing decrease in community engagement results from a cyclical process of neighborhood depopulation, structural neglect, property vacancy, fear of crime, and crime itself (Garvin et al., 2012; Sampson et al., 2017). Residents in this study readily shared ideas for abandoned property repurposing, demonstrating that community input is crucial to implementing locally and culturally relevant neighborhood improvement interventions. Such programs have been shown to have a more significant effect, particularly on crime reduction, when programs engage residents in the efforts (Rupp et al., 2022; Gong et al., 2022). Similarly expressed by participants, research has shown that resident involvement in neighborhood maintenance likely increases connectedness by encouraging prosocial interactions and providing visual markers that neighbors are watchful and invested in their surroundings (Gong et al., 2022). Such an approach may be essential in disinvested communities where community members often express low confidence in local government to maintain neighborhood infrastructure, as frequently stated by residents in this study.

Third, residents shared detailed accounts of community characteristics that inform their sense of safety, often speaking about signs of property neglect and varying levels of social engagement among neighbors. A group of themes emerging from this study indicate residents’ perspectives on the importance of interpersonal relationships in neighborhood environments acting as a mediator of individual sense of community and safety from crime and physical hazards. These themes include *neighborhood interpersonal relationships cultivate a sense of community and support systems; the presence of community crime influences perceptions of personal safety, social engagement, and health; neighborhood social networks as crime prevention; positive youth engagement in the home and community as crime reduction and prevention strategies.* These themes support the routine activity theory that posits attentive neighbors informally act as “capable guardians” who can intentionally or inadvertently mitigate neighborhood crime (Cohen and Felson, 1979). Sense of safety, as spoken about by residents, upholds observed connections between neighborhood physical disorder (i.e., unkempt conditions) and social disorder (i.e., crime, weak social ties) as related to collective efficacy (Sampson and Raudenbush, 1999). The theme, *neighborhood social networks as crime prevention,* demonstrates that resident collective efficacy supports resident sense of safety, including hazards that may result from physical disorder. Research conducted by Pearson et al., (2021, 2018) suggested that a lower sense of safety, combined with weak social ties and property vacancy, is linked to greater stress and adverse health outcomes.

Moreover, residents often spoke of increased hypervigilance, which has been shown to be connected to adverse cognitive and behavioral outcomes that impact broader community health (Smith et al., 2019). Also, feeling safe walking around alone in one’s neighborhood is critical for promoting an active lifestyle that advances health and well-being (Robinette et al., 2016). Similarly, qualitative photovoice essays led by Sampson et al., (2017) found that participants demonstrated that residential connection to the community highly impacted collective efficacy, physical, and mental health.

A final theme of importance posits *positive youth engagement in the home and community as crime reduction and prevention strategies*. Youth and emerging adult participants discussed how they felt particularly vulnerable to violence, especially amongst Black, gender non-conforming youth and girls, corroborating evidence that these populations are at greater risk of violence (*Preventing Youth Violence |Violence Prevention|Injury Center|CDC*, n.d.-b). Adult participants primarily focused on the need for positive youth engagement in the home and community as a crime reduction and prevention strategy. Positive youth engagement is a practical approach to addressing youth violence across multiple settings (e.g., home, school, and neighborhood) and interpersonal relationships (e.g., caregivers, peers, educators) (Catalano et al., 2004). Studies on the portrayal of youth violence in print media have shown that news outlets and consumers tend to vilify and emphasize individual-level solutions for youth offenders instead of modifying structural causes and environmental moderators of violence (McManus and Dorfman, 2002). However, as expressed by participants and existing literature, community-level factors such as the physical environment, concentrated poverty, residential instability, the density of alcohol outlets, and social norms, are critical in preventing and mitigating youth violence (Kingston et al., 2021).

This study is strengthened by its large and demographically diverse sample of New Orleans residents, investigating how neglected properties and neighborhood environmental characteristics influence resident outcomes. This study has limitations that should be addressed. First, we recruited residents and identified key informant participants through community-based organizations, resulting in snowball and convenience sampling, which limits the results’ generalizability. Second, COVID-19 safety measures resulted in an amended data collection protocol to complete interviews online using Zoom. There were instances where participants experienced difficulty navigating the software, and unstable internet connectivity impacted the quality of the data collected; additionally, two interview recordings were lost due to incomplete download, and the interview notes were used for analysis. Nevertheless, most interviews conducted over Zoom proved to be a successful alternative, allowing the study team to adhere to safety measures, continue to ensure participant confidentiality and participation preferences (e.g., ability to use the chat feature or be off camera), meet participants’ availability, and remove the need for transportation and meeting logistics.

## Conclusion

The impact of environmental conditions on health outcomes in any given community is multifaceted and far-reaching. Our findings contribute a rich understanding of the ways in which residents perceive and are socially, physically, and behaviorally affected by neighborhood environment characteristics and the presence of neglected conditions. Participants in our study emphasized the importance of community-level interventions in long-neglected physical environments within urban neighborhoods to reduce violence, increase safety and community participation, and amplify markers of community well-being. Golden and Earp, (2012) discussed the popularity of ecological models in public health discourses; however, the extent to which comprehensive approaches are adopted and sustained in practice is limited. Engaging residents, especially youth, about their lived experiences and narratives related to their neighborhood environment provides an opportunity to develop community-informed solutions that counter the negative impacts of built environment neglect. This study situates residents' local experiences, knowledge, and preferences as a critical element in a multi-method study (i.e., The Healthy Neighborhoods Project) to better inform policy and practice in New Orleans and other urban communities seeking environmental-level solutions to improve resident well-being.

## Data Availability

The raw data analyzed for this study are not publicly available to preserve participant privacy. Deidentified data may be made available upon request.
